# Radiomics nomogram for the preoperative prediction of lymph node metastasis in pancreatic ductal adenocarcinoma

**DOI:** 10.1186/s40644-021-00443-1

**Published:** 2022-01-06

**Authors:** Yun Bian, Shiwei Guo, Hui Jiang, Suizhi Gao, Chengwei Shao, Kai Cao, Xu Fang, Jing Li, Li Wang, Chao Ma, Jianming Zheng, Gang Jin, Jianping Lu

**Affiliations:** 1 Department of Radiology, Changhai Hospital, The Naval Medical University, 168 Changhai Road, Shanghai, 200433 China; 2 Department of Pancreatic Surgery, Changhai Hospital, The Naval Medical University, Shanghai, China; 3 Department of Pathology, Changhai Hospital, The Naval Medical University, Shanghai, China

**Keywords:** Pancreatic neoplasm, Carcinoma, Pancreatic ductal adenocarcinoma, Lymph nodes, Computed tomography, Radiomics, Nomograms

## Abstract

**Purpose:**

To develop and validate a radiomics nomogram for the preoperative prediction of lymph node (LN) metastasis in pancreatic ductal adenocarcinoma (PDAC).

**Materials and methods:**

In this retrospective study, 225 patients with surgically resected, pathologically confirmed PDAC underwent multislice computed tomography (MSCT) between January 2014 and January 2017. Radiomics features were extracted from arterial CT scans. The least absolute shrinkage and selection operator method was used to select the features. Multivariable logistic regression analysis was used to develop the predictive model, and a radiomics nomogram was built and internally validated in 45 consecutive patients with PDAC between February 2017 and December 2017. The performance of the nomogram was assessed in the training and validation cohort. Finally, the clinical usefulness of the nomogram was estimated using decision curve analysis (DCA).

**Results:**

The radiomics signature, which consisted of 13 selected features of the arterial phase, was significantly associated with LN status (*p* < 0.05) in both the training and validation cohorts. The multivariable logistic regression model included the radiomics signature and CT-reported LN status. The individualized prediction nomogram showed good discrimination in the training cohort [area under the curve (AUC), 0.75; 95% confidence interval (CI), 0.68–0.82] and in the validation cohort (AUC, 0.81; 95% CI, 0.69–0.94) and good calibration. DCA demonstrated that the radiomics nomogram was clinically useful.

**Conclusions:**

The presented radiomics nomogram that incorporates the radiomics signature and CT-reported LN status is a noninvasive, preoperative prediction tool with favorable predictive accuracy for LN metastasis in patients with PDAC.

**Supplementary Information:**

The online version contains supplementary material available at 10.1186/s40644-021-00443-1.

## Introduction

Pancreatic cancer is a highly lethal disease, and its mortality closely parallels its incidence [1, 2]. Surgical resection is regarded as the only potentially curative treatment and can result in significantly longer survival than other treatment options. Lymph node (LN) metastases are observed in 70% or more of resected ductal adenocarcinomas and are present even when the primary tumor is small (< 2 cm) [3]. LN variables remain some of the most important individual predictors of survival. A reliable method to obtain accurate LN results is postoperative pathology. However, this technique is limited in detecting LN metastasis during preoperative staging.

Endoscopic ultrasonography-guided fine-needle aspiration (EUS-FNA) is considered quite sensitive for detecting LN metastases from pancreatic lesions, but EUS-FNA is an invasive diagnostic tool that is expensive and time consuming and has a rather significant risk of complications [4, 5]. Several factors limit the use of magnetic resonance imaging (MRI) for determining LN status in clinical cohort settings, including spatial resolution problems, motion artifacts and dose-dependent oversaturation artifacts [6]. Multislice computed tomography (MSCT) is the best initial diagnostic test for pancreatic cancer. However, a meta-analysis that investigated CT for assessing extraregional LN metastases in pancreatic and periampullary cancer yielded a pooled sensitivity of 25% and a positive of predictive value (PPV) of 28% [7]. Important clinical objectives, including differentiation of reactive, inflammatory lymphadenopathy from malignant lymphadenopathy and detection/visualization of micrometastases, were not achieved with this technique.

Radiomics is an emerging field that converts imaging data into a high-dimensional mineable feature space using a large number of automatically extracted data characterization algorithms [8, 9]. Radiomics provides a noninvasive method for the prediction of LN metastasis. At present, there are few studies on predicting LN metastasis using radiomics [10–13]. To the best of our knowledge, no studies have determined whether a radiomics signature would enable superior prediction of LN metastasis from PDAC.

Therefore, in this present study, we aimed to develop and validate a radiomics nomogram incorporating a radiomics signature and CT-reported LN status for the preoperative prediction of LN metastasis in patients with PDAC.

## Patients and methods

### Patients

**Fig. 1:**
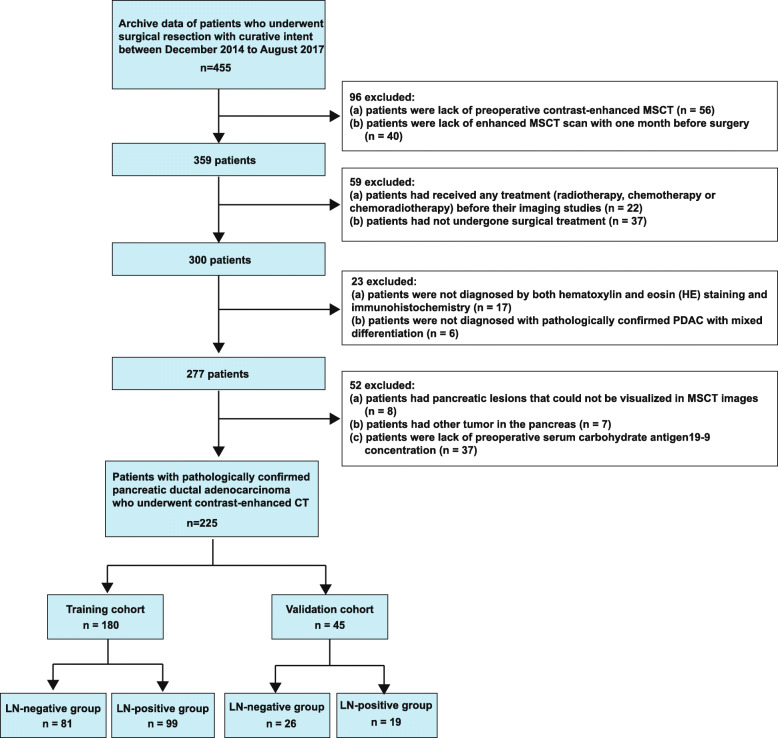
The patient enrolment process for this study

This retrospective single-center study was reviewed and approved by the Biomedical Research Ethics Committee of the Navy Military Medical University of the Chinese People’s Liberation Army. Patients were excluded from the study if one of the following criteria was met: patients who had not undergone preoperative standard contrast-enhanced MSCT, had not undergone enhanced MSCT within a month before surgery, had received any treatment (radiotherapy, chemotherapy or chemoradiotherapy) before undergoing imaging studies, had not undergone surgery, were not diagnosed with PDAC by both hematoxylin and eosin (HE) staining and immunohistochemistry, had pathologically confirmed PDAC with mixed differentiation, had pancreatic lesions that could not be visualized by MSCT, had other tumors in the pancreas, or lacked preoperative serum carbohydrate antigen 19–9 (CA 19–9) concentration. Consequently, a total of 225 consecutive patients with PDAC, 137 males (mean age, 60.02 years; age range, 31–77 years) and 88 females (mean age, 63.28 years; age range, 32–80 years), were included in this cross-sectional study at our institution. A flowchart of the study population is presented in Fig. 1. We divided the patients into two independent cohorts. One hundred eighty consecutive patients constituted a training cohort of 107 males (mean age, 59.19 years; age range, 31–75 years) and 73 females (mean age, 62.59 years; age range, 32–75 years). Data were gathered from records between January 2014 and January 2017. Forty-five consecutive patients constituted a validation cohort of 30 males (mean age, 63.00 years; age range, 45–77 years) and 15 females (mean age, 64.13 years; age range, 46–80 years). Data were gathered from records between February 2017 and December 2017.

### CT scanning

A 640-slice CT scanner (Aquilion ONE, Canon Medical Systems, Tokyo, Japan) was used with the following CT scan parameters: 120 kV, 150 effective mAs, beam collimation of 160 × 0.5 mm, a matrix of 350 × 350, and a gantry rotation time of 0.5 s. After nonenhanced CT scanning, dynamic contrast-enhanced CT scanning was performed. The scan delayed time was determined according to the test bolus. The contrast agent, 90–95 mL of 370 mgI/mL iopromide (Ultravist 370, Bayer Healthcare, Berlin, Germany), was injected at a rate of 5.5 ml/sec with a power injector (Medrad Mark V plus, Bayer, Leverkusen, Germany) via the forearm vein, followed by 98 ml of normal saline to flush the tube. Arterial (20–25 s), portal venous (60–70 s), and delayed-phase (110–130 s) scans were performed after contrast agent injection. The slice thickness/intervals of the scan and reconstruction were 0.8/1.0 mm and 1.0/1.0 mm, respectively. The scanning range was from the level of the diaphragm to the level of the pelvis.

### Imaging analysis

All CT images were analyzed by two board-certified abdominal radiologists (W.L. and F.X., with 30 and 5 years of experience, respectively) who were aware that the study population had PDAC but were blinded to the clinical and pathologic details.

All tumors were evaluated for the following 6 features: (a) Tumor location was defined as in the head, body, or tail of the pancreas or in multiple locations in the pancreas. (b) Tumor size was defined as the maximum diameter of a cross-section of the tumor [14]. (c) CT-reported LN metastasis was considered if one of the following six criteria was met: short-axis diameter of a LN > 10 mm, nonuniform density, nonuniform enhancement, internal necrosis, LN fusion, ill-defined borders, or involvement of surrounding organs or blood vessels [15, 16]. (d) Organ invasion was defined as involvement of the liver, spleen, intestines, or stomach in which the tumor could not be separated from the organs. (e) Vascular invasion was defined as invasion of the common hepatic artery, splenic artery and vein, gastroduodenal artery, superior mesenteric artery and vein, or portal vein. The criteria for vascular invasion included vessel occlusion or stenosis or tumor contacting more than half of the perimeter of the vessel.

### Radiomics workflow

**Fig. 2:**
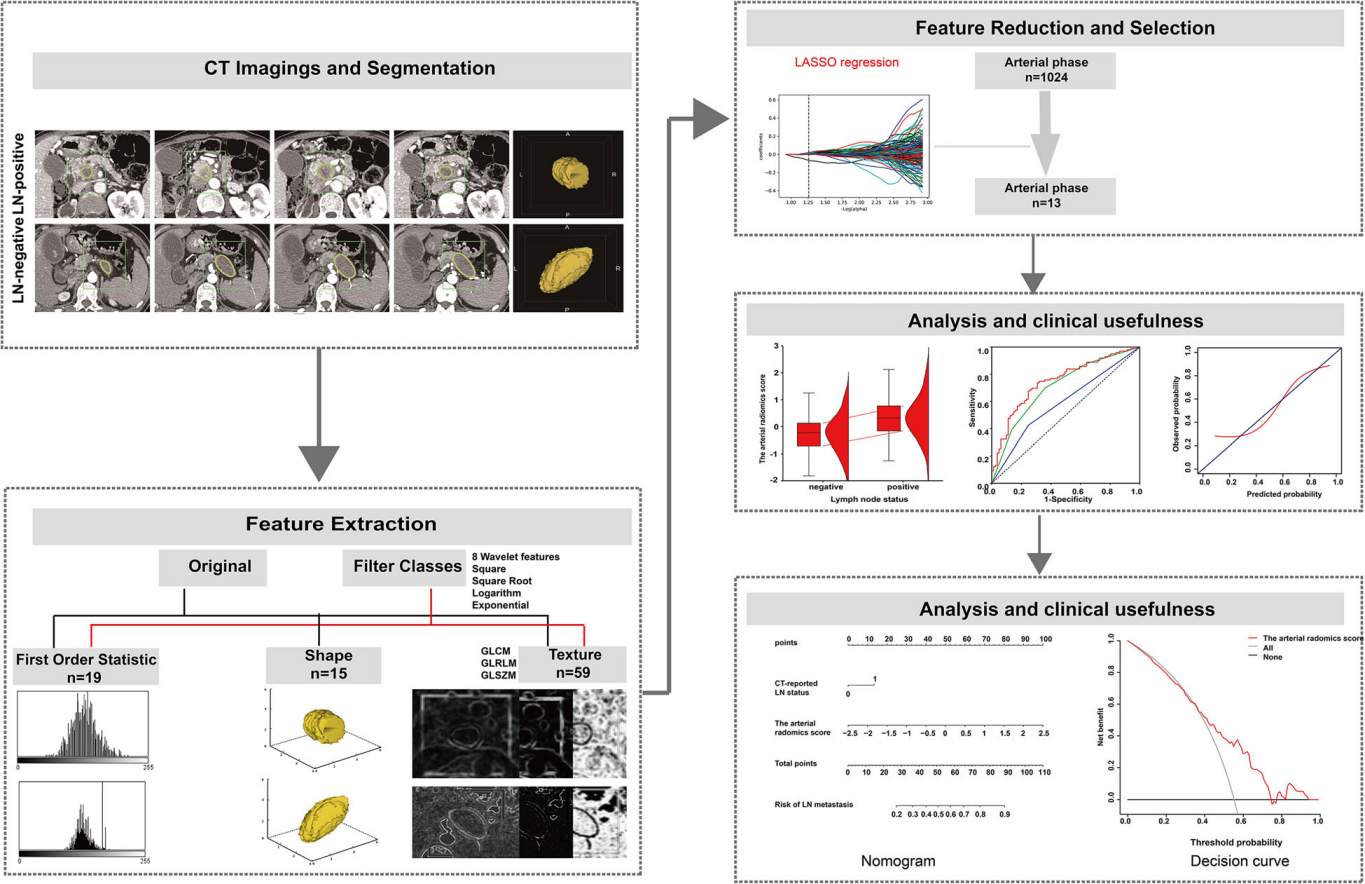
Radiomics workflow

The radiomics workflow included (a) image segmentation, (b) feature extraction, (c) feature reduction and selection, and (e) predictive model building (Fig. 2).

### Image segmentation, Radiomics feature extraction and Radiomics signature building

In this study, radiomics features were extracted from arterial CT scans. The draw tool available in the Editor module of 3D Slicer version 4.8.1 (open source software: https://www.slicer.org/) was used to delineate the tumors in multiple slices. In this study, the volume of interest was extracted by stacking the corresponding regions of interest (ROIs) delineated slice-by-slice for each patient. Image preprocessing can be found in Supplementary 1.

Radiomics feature extraction was conducted using an open source Python package, PyRadiomics 1.2.0 (http://www.radiomics.io/pyradiomics.html) [17]. The feature extraction methods used in this study included two categories: original feature classes and filter classes. The filter classes further included five categories: wavelet, square, square root, logarithm, and exponential. A total of 1029 2D and 3D features from primary tumors in the arterial phase were extracted and divided into five groups: (a) first-order statistics, (b) shape features, (c) gray-level cooccurrence matrix (GLCM) features, (d) gray-level size zone matrix (GLSZM) features, and (e) gray-level run-length matrix (GLRLM) features. More information about the procedures for image segmentation and radiomics feature extraction is reported in Supplementary 2.

To assess interobserver reliability, the ROI segmentation was performed in a blinded fashion by two radiologists: reader 1 (W.L.) and reader 2 (F.X.). To evaluate intraobserver reliability, reader 1 repeated the feature extraction 3 times at the interval of 1 week. Reader 1 completed the remaining image segmentations, and the readout sessions were conducted over a period of 1 month. The reliability was calculated by using intraclass correlation coefficients (ICCs). Radiomics features with both interobserver and intraobserver ICC values greater than 0.75 (indicating excellent stability) were selected for subsequent investigation.

As the radiomics features were very high-dimensional compared with the sample size, the least absolute shrinkage and selection operator (LASSO) logistic regression algorithm, suitable for performing regression analysis of high-dimensional data, was used to select the most useful associated features [18]. The LASSO logistic regression model was used with penalty parameter tuning that was conducted by 5-fold cross-validation based on minimum criteria. The radiomics score (rad-score) was calculated for each patient via a linear combination of selected features weighted by their respective coefficients. More information about feature selection can be found in Supplementary 3.

### Development, performance, and validation of a Radiomics model

Multivariable logistic regression analysis was conducted to develop a model for predicting LN metastasis in the primary cohort. To provide a more understandable outcome measure, a nomogram was then constructed by using the selected covariates. The discrimination performance of established models was quantified by the receiver operating characteristic (ROC) curve, which was constructed using Bootstrap resampling (times = 500), and the area under the curve (AUC) value [19]. AUC estimates in the prediction models were compared by using the Delong nonparametric approach [20]. Calibration curves was plotted via bootstrapping with 500 resamples to assess the calibration of the radiomics model, accompanied by the Hosmer-Lemeshow goodness-of-fit test. The performance of the radiomics model was then internally tested in an independent validation cohort by using the formula derived from the primary cohort.

### Clinical utility of the Radiomics nomogram

To estimate the clinical utility of the nomogram, decision curve analysis (DCA) was performed by calculating the net benefits for a range of threshold probabilities (Supplementary 4).

### Pathological image analysis

All the specimens were analyzed by a specialized pathologist. Pathological examinations and analyses were standardized according to a formal protocol [21]. The resected specimens were immediately fixed in formalin for 24 h. Subsequently, they were cut horizontally into 5-mm tissue blocks that were dehydrated and embedded in paraffin. Finally, 5-μm sections were stained with HE for conventional histology. Each large section was carefully examined by light microscopy. Tumor–node–metastasis (TNM) staging was performed on the basis of the American Joint Committee on Cancer TNM Staging Manual, 8th Edition [15].

### Statistical analysis

Normal distribution and variance homogeneity tests were performed on all continuous variables. Continuous variables with a normal distribution are expressed as mean ± SD; variables with a non-normal distribution are expressed as the median and interquartile range. The rad-score was expressed as ten times. First, we examined group differences in terms of age, gender, body mass index (BMI), CA 19–9 level, tumor location, tumor (T) grade, differentiation grade, and the rad-score between the LN-positive and LN-negative patients. Student’s t test (normal distribution), the Kruskal-Wallis H (skewed distribution) test, and the chi-square test (categorical variables) were used to identify significant differences between the two groups. Second, patients were categorized into quartiles (Q1 < -0.45, Q2 [− 0.45 to − 0], Q3 [0 to 0.46], and Q4 ≥ 0.46) on the basis of the rad-score, with Q1 as the reference group. Univariate regression analysis was applied to estimate the effect sizes between all variables and LN metastasis. Variables that reached statistical significance in the univariable analysis were considered for the multivariable model.

A two-tailed *p*-value less than 0.05 was considered statistically significant. All analyses were performed with R (R version 3.3.3; R Foundation for Statistical Computing; http://www.r-project.org) and EmpowerStats (X&Y Solutions, Inc., Boston, MA, USA).

## Results

### Clinical characteristics

The LN-negative and LN-positive patients accounted for 47.56% (107) and 52.44% (118) of the study cohort, respectively. There was a significant difference in M stage between the LN-positive and LN-negative patients in the training cohort (*p* = 0.043). However, there were no significant differences in age, gender, CA 19–9 level, T stage, M stage of the validation cohort or differentiation grade (*p* > 0.05) between the 2 groups. The patient characteristics are shown in Table 1.

**Table 1:** Baseline and MSCT Characteristics of Patients with PDAC

Characteristics	Training cohort			Validation cohort		
	LN-negative (*n* = 81)	LN-positive (*n* = 99)	*P* value	LN-negative (*n* = 26)	LN-positive (*n* = 19)	*P* value
Age (y, mean ± SD)	61.7 ± 7.2	59.6 ± 9.8	0.115	62.9 ± 8.1	64.1 ± 7.3	0.620
BMI (kg/m^2^, mean ± SD)	22.6 ± 2.8	22.6 ± 2.6	0.999	23.17 ± 3.0	22.5 ± 2.4	0.422
Sex						
Male	47 (58.0)	60 (60.6)	0.726	16 (61.5)	14 (73.7)	0.393
Female	34 (42.0)	39 (39.4)		10 (38.5)	5 (26.3)	
CA19–9 (μg/L, median, min-max)	332.1 (71.6–1066.6)	293.5 (103.7–1200.0)	0.479	278.4 (13.6–1200.0)	305.5 (41.8–1200.0)	0.926
CA 19–9						
< 1000 μg/L	57 (70.4)	65 (65.7)	0.501	17 (65.4)	13 (68.4)	0.831
≥1000 μg/L	24 (29.6)	34 (34.3)		9 (34.6)	6 (31.6)	
T stage						
T1	14 (17.3)	8 (8.1)	0.158	5 (19.2)	0	0.157
T2	15 (18.5)	23 (23.2)		5 (19.2)	5 (26.3)	
T3–4	52 (64.2)	68 (68.7)		16 (61.5)	14 (73.7)	
M stage						
M0	80 (98.8)	91 (91.9)	0.043	23 (88.5)	19 (100.0)	0.068
M1	1 (1.23)	8 (8.1)		0	3 (11.5)	
Differentiation grade						
Well to moderately	65 (80.3)	85 (85.9)	0.315	23 (88.5)	12 (63.2)	0.070
Poorly to undifferentiated	16 (19.8)	14 (14.14)		3 (11.5)	7 (36.8)	
Surgery						
Pylorus-preserving pancreatoduodenectomy	2 (2.47)	10 (10.1)	0.133	2 (7.7)	2 (10.5)	0.781
Pancreatoduodenectomy	43 (53.1)	53 (53.5)		14 (53.9)	9 (47.4)	
Total pancreatectomy	7 (8.6)	4 (4.0)		1 (3.9)	0	
Distal pancreatectomy	29 (35.8)	32 (32.3)		9 (34.6)	8 (42.1)	
Location						
Head	44 (54.3)	63 (63.6)	0.205	16 (61.5)	11 (57.9)	0.805
Neck, body and tail	37 (45.7)	36 (36.4)		10 (38.5)	8 (42.1)	
Size (mm, median, interquartile range)	26.0 (19.8–31.2)	23.5 (19.0–31.8)	0.885	24.4 (16.7–28.9)	29.2 (21.3–35.3)	0.044
Vascular invasion						
No	50 (61.7)	60 (60.6)	0.878	19 (73.1)	11 (57.9)	0.286
Yes	31 (38.3)	39 (39.4)		7 (26.9)	8 (42.1)	
Organ invasion						
No	72 (88.9)	86 (86.9)	0.681	22 (84.6)	15 (78.0)	0.704
Yes	9 (11.1)	13 (13.1)		4 (15.4)	4 (21.1)	
CT-reported LN status						
Negative	61 (75.3)	57 (57.6)	0.013	19 (73.1)	9 (47.4)	0.079
Positive	20 (24.7)	42 (42.4)		7 (26.9)	10 (52.6)	

Data are presented as n (%)

*MSCT* multislice computed tomography, *LN* lymph node, *CA19–9* carbohydrate antigen 19–9

### Tumor MSCT features

Among various CT findings, tumor size in the validation cohort and CT-reported LN status in the training cohort differed significantly between the LN-negative and LN-positive patients. However, there were no significant differences in tumor location, tumor size in the training cohort, vascular invasion, organ invasion, and CT-reported LN status in the validation cohort between the LN-negative and LN-positive patients (Table 1).

### Radiomics analysis

A total of 1029 radiomics features from the arterial phase of CT were extracted and grouped on the basis of LN metastasis. We removed the 480 radiomics feature with ICC values < 0.75. The interobserver ICCs of the 549 radiomics features were good, ranging from 0.80 to 0.91. The intraobserver ICCs of the 549 radiomics features were also good, ranging from 0.86 to 0.92. Next, the radiomics features that did not significantly different between the groups or did not show significant correlations with LN-positive/negative was excluded. The 24 remaining radiomics features were further reduced using a LASSO logistic regression model. Finally, the radiomics characteristics were reduced to 13 features. Finally, the radiomics signature was constructed, and the radiomics scores was calculated by using the following formula 1. There was a significant difference in arterial rad-score between the LN-positive and LN-negative patients (*p* = 0.002) (Supplementary 5).

Formula 1

Radiomics score = 0.52444–0.00008 × original.shape. Compactness1–0.06501 × original.shape. Compactness2 + 0.01910 × original.glrlm. LowGrayLevelRunEmphasis + 0.00487 × original.glrlm. LongRunLowGrayLevelEmphasis

+ 0.01580 × logarithm.glszm. GrayLevelNonUniformityNormalized

+ 0.01921 × square.firstorder. Skewness

- 0.00490 × exponential.glszm. GrayLevelNonUniformityNormalized

+ 0.01238 × wavelet- LHL.firstorder. Mean

+ 0.00024 × wavelet-LHL.firstorder. Kurtosis

+ 0.00282 × wavelet-HLL.firstorder. Mean

+ 0.01832 × wavelet - LLH.glszm. LargeAreaHighGrayLevelEmphasis

+ 0.00264 × wavelet-HLH.glrlm. LowGrayLevelRunEmphasis

+ 0.01276 × wavelet - LLL.firstorder. Mean

### Univariate analysis of each parameter

The univariate analysis results are shown in Table 2. The rad-score (*p* < 0.0001) and CT-reported LN status (*p* = 0.014) were significantly associated with an increased risk for LN metastasis.

**Table 2:** Results of univariate analysis in the training cohort for predicting LN metastasis

Variables	Statistics	Odds Ratio (95% CI)	*P* value
Rad-score (median, interquartile range)	0 (− 0.45–0.46)	3.25 (1.99, 5.33)	< 0.0001
Rad-score			
Q1	41 (22.8)	1.0 (reference)	
Q2	41 (22.8)	1.89 (0.76, 4.71)	0.1712
Q3	48 (26.8)	4.03 (1.65, 9.82)	0.0022
Q4	50 (27.8)	8.57 (3.32, 22.13)	< 0.0001
Age (y, mean ± SD)	60.5 + 8.8	0.97 (0.94, 1.01)	0.1167
BMI (kg/m^2^, mean ± SD)	22.6 + 2.7	1.00 (0.90, 1.12)	0.9987
Sex			
Male	107 (59.4)	1.0 (reference)	
Female	73 (40.6)	0.90 (0.49, 1.63)	0.7257
CA 19–9			
< 1000 μg/L	122 (67.8)	1.0 (reference)	
≥1000 μg/L	58 (32.2)	1.24 (0.66, 2.34)	0.5011
Location			
Head	107 (59.4)	1.0 (reference)	
Neck, body and tail	73 (40.6)	0.68 (0.37, 1.24)	0.2062
T grade			
T1	22 (12.2)	1.0 (reference)	
T2	38 (21.1)	2.68 (0.91, 7.94)	0.0746
T3–4	120 (66.7)	2.29 (0.89, 5.86)	0.0845
Differentiation grade			
Well to moderately	150 (83.3)	1.0 (reference)	
Poorly to undifferentiated	30 (16.7)	0.67 (0.30, 1.47)	0.3168
Size (mm, median, interquartile range)	25.01 (19.0–31.6)	1.01 (0.98, 1.04)	0.5712
Vascular invasion			
No	110 (61.1)	1.0 (reference)	
Yes	70 (38.9)	1.05 (0.57, 1.92)	0.8779
Organ invasion			
No	158 (87.8)	1.0 (reference)	
Yes	22 (12.2)	1.21 (0.49, 2.99)	0.6809
CT-reported LN status			
Negative	118 (65.6)	1.0 (reference)	
Positive	62 (34.4)	2.25 (1.18, 4.28)	0.0136

Data are presented as n (%)

*Rad-score* radiomics score, *LN* lymph node, *CA19–9* carbohydrate antigen 19–9, *CI* confidence interval

Patients were categorized into quartiles by radiomics score (Q1 < -0.45, Q2 [−0.45 to 0], Q3 [0 to 0.46], and Q4 ≥ 0.46)

### Development, performance, and validation of prediction models

**Fig. 3:**
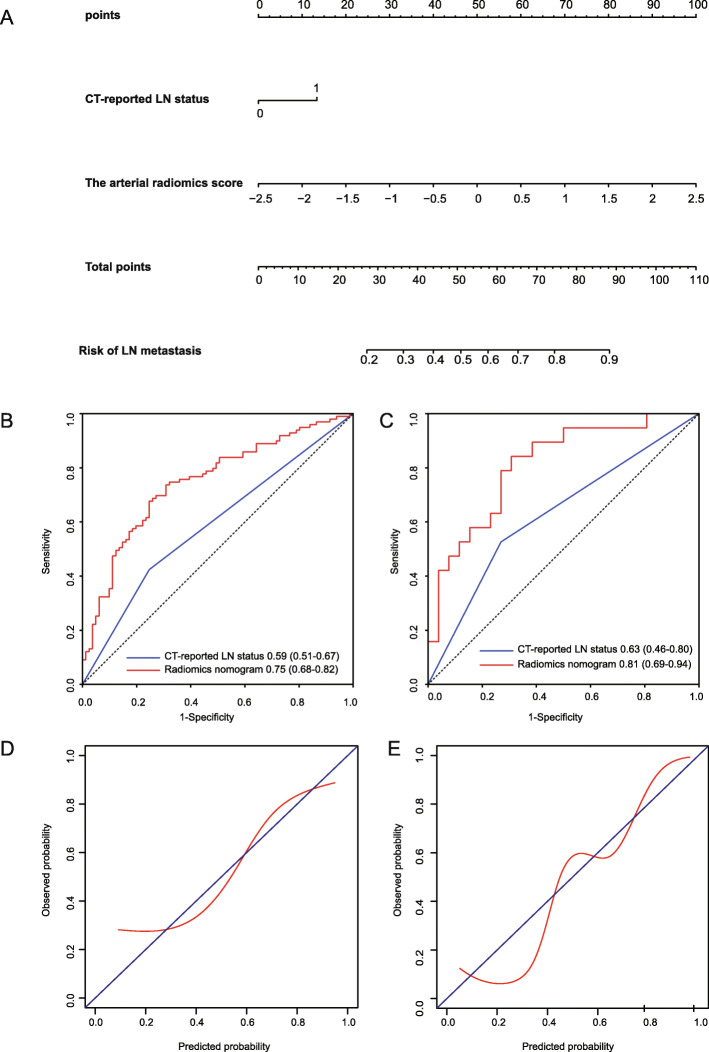
Radiomics nomogram developed with ROC and calibration curves. **A** A radiomics nomogram was developed in the primary cohort, incorporating the radiomics signature and CT-reported LN status. Comparison of ROC curves between the radiomics nomogram and CT-reported LN status alone for the prediction of LN metastasis in the (**B**) primary and (**C**) validation cohorts. Calibration curves of the radiomics nomogram in the (**D**) primary and (**E**) validation cohorts

Logistic regression analysis identified the rad-score and CT-reported LN status as independent predictors (Table 3). A model that incorporated these two independent predictors was developed and presented as a nomogram (Fig. 3A). In addition, the prediction model was built based on CT-reported LN status.

**Table 3:** The multivariable logistic regression model for LN metastasis of PDAC

Variable	Coefficient	S.E.	OR (95% CI)	*P*-value
(Intercept)	−0.10	0.20	0.91 (0.61, 1.34)	0.62
CT-reported LN metastasis				
No versus yes	0.79	0.35	2.19 (1.10, 4.38)	0.026
Rad-score	1.17	0.26	3.24 (1.96, 5.35)	0.0001

The predicted model = −0.10 + 0.79× (CT-reported LN metastasis =1) + 1.17 × Rad-score

*Note*: *S. E.* standard error, *OR* odds ratio, *CI* confidence interval, *LN* lymph node, *Rad-score* radiomics score

All ROC curves are provided in Fig. 3B and 3C. In the primary cohort, the radiomics model showed the highest discrimination between LNs that were positive and negative for metastasis, with an AUC of 0.75 (95% CI: 0.68, 0.82); the observed AUC value was higher than that for CT-reported LN status (AUC, 0.59 [95% CI: 0.51, 0.67]; *p* < 0.0001). In the validation cohort, the radiomics model yielded the greatest AUC (0.81; 95% CI: 0.69, 0.94), which confirmed that the radiomics model achieved better predictive efficacy than CT-reported LN status (AUC, 0.63 [95% CI: 0.46, 0.80]; *p* = 0.02). In the radiomics model, the sensitivity, specificity, and accuracy for the training cohort were 67.68, 75.31%, and 0.711, respectively, whereas those for the validation cohort were 84.21, 69.23%, and 0.756, respectively. The calibration curve of the radiomics nomogram demonstrated good agreement between predicted and observed LN metastasis in the primary cohort (Fig. 3D). The Hosmer-Lemeshow test yielded a *p-*value of 0.88, suggesting no departure from the good fit. The favorable calibration of the radiomics nomogram was further confirmed in the validation cohort (Fig. 3E). The Hosmer-Lemeshow test yielded a p*-*value of 0.63, suggesting a perfect fit of the nomogram in the validation set.

### Clinical use

**Fig. 4:**
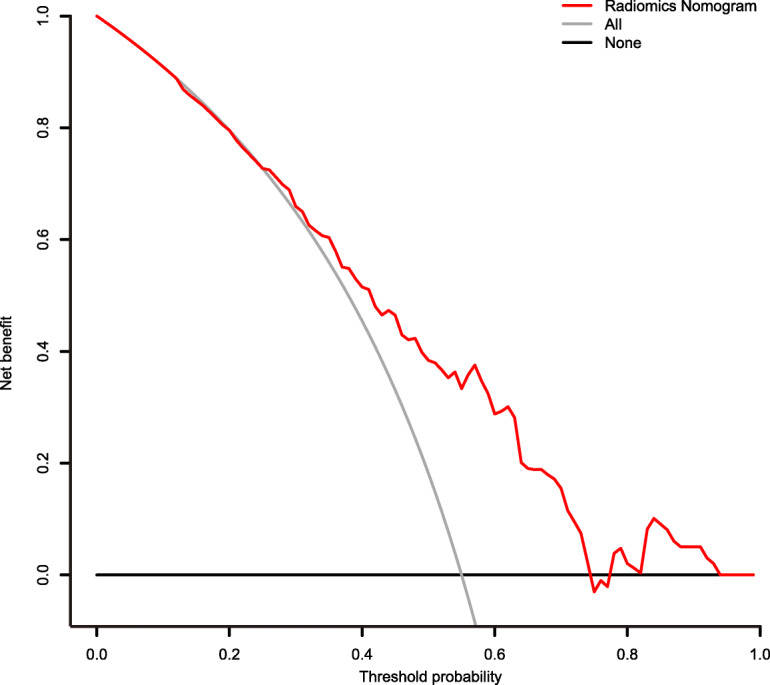
DCA for the rad-score. DCA for the radiomics nomogram. The y-axis represents the net benefit. The red line represents the radiomics nomogram. The gray line represents the hypothesis that all patients had LN metastases. The black line represents the hypothesis that no patients had LN metastases. The x-axis represents the threshold probability, which is where the expected benefit of treatment is equal to the expected benefit of avoiding treatment. The decision curves in the validation set showed that if the threshold probability is between 0.25 and 0.75, the radiomics nomogram developed in the current study to predict LN metastases adds more benefit than the treat-all or treat-none scheme

The DCA in the validation set showed that if the threshold probability is between 0.25 and 0.75, using the radiomics nomogram in the current study to predict LN metastases adds more benefit than the treat-all-patients scheme or the treat-none scheme (Fig. 4).

## Discussion

We developed and validated a diagnostic, radiomics signature-based nomogram for the preoperative individualized prediction of LN metastasis in patients with PDAC. The nomogram incorporates two items, the rad-score and CT-reported LN status. Incorporating these two factors into an easy-to-use nomogram facilitates the preoperative individualized prediction of LN metastasis.

PDAC is characterized by an extremely high mortality and a poor prognosis, which are largely attributed to difficulties in early diagnosis and limited therapeutic options. The number of positive LNs has been shown to be a crucial and independent prognostic factor for overall survival in PDAC [22]. Pancreatectomy is the most effective method to improve long-term patient survival. Whether pancreatectomy should include standard and extended lymphadenectomy is still debated [23, 24]. Accurate preoperative LN staging of PDAC is essential for providing patients with appropriate counsel regarding surgical decisions and prognosis. However, it is difficult with the currently available methods.

TH high-risk patients should consider neoadjuvant therapy if LN metastases are confirmed by endoscopic ultrasonography-guided FNA (EUS-FNA) [25]. EUS-FNA is considered quite sensitive for the detection of pancreatic lesions and offers diagnostic value for both the primary tumor and LN metastases [4, 5]. A piece of tissue that can provide sufficient histological information to help diagnose peripancreatobiliary LN involvement can be obtained with EUS-FNA. For FNA of LNs, suction is not recommended to reduce blood contamination [26]. In addition, EUS-FNA is affected by various factors, such as scope position [27], lesion characteristics, the environment surrounding the lesions, and the evaluating pathologist [27–30]. Positron emission tomography–computed tomography (PET/CT) is limited in its ability to evaluate small lesions and cannot differentiate between inflammatory lymphadenopathy and metastatic lymphadenopathy [31]. Similarly, MRI has several limiting factors associated with the determination of LN status in clinical settings, namely, spatial resolution problems, motion artifacts, and dose-dependent oversaturation artifacts [6]. The most widely used preoperative staging modality for pancreatic cancer is CT [32, 33], which can accurately assess tumor size and vessel involvement, but its diagnostic accuracy for adequately assessing LN involvement is limited. Many studies have reported a diagnostic sensitivity of only 20–38% [34–37]. In the current study, the AUCs of CT-reported LN status were only 0.59 (95% CI, 0.51 to 0.67) in the training cohort and 0.63 (95% CI, 0.46 to 0.80) in the validation cohort.

There are several main limitations of preoperative imaging studies of LNs. First, LN imaging findings are difficult to correlate one-to-one with pathological evidence of LN metastasis. Second, CT has limited visualization ability to identify metastatic LNs. Finally, there is no significant correlation between LN metastasis and the clinical and pathologic characteristics of PDAC patients. In addition, local inflammation secondary to malignant biliary obstruction may independently result in enlarged LNs [38]. In the current study, we found no significant correlation between LN metastasis and CT-reported tumor size or vascular or organ invasion. Thus, improved predictive tools for preoperative LN staging are urgently needed. In our study, the arterial radiomics signature was significantly associated with LN status (*p* < 0.05 for both the training and validation cohorts).

At present, there are few studies on predicting LN metastasis using radiomics. Wu et al. [10] developed and validated a radiomics nomogram that incorporated the radiomics signature and CT-reported LN status and showed good calibration and discrimination in a training set (AUC, 0.9262; 95% CI, 0.8657–0.9868) and in a validation set (AUC, 0.8986; 95% CI, 0.7613–0.9901). Huang et al. [12] developed and validated a radiomics nomogram that included the radiomics signature, carcinoembryonic antigen (CEA) level and CT-reported LN status, and the prediction model yielded C-indexes of 0.736 (95% CI, 0.730 to 0.742) in the training cohort and 0.778 (95% CI, 0.769 to 0.787) in the validation cohort. A nomogram incorporating some clinical and pathological factors to predict the prognosis of PDAC has been reported [39, 40]. However, it was difficult to incorporate the radiomics signature, imaging findings and clinical factors to predict LN metastasis from PDAC. In the current study, the rad-score and CT-reported LN status were incorporated into an easy-to-use nomogram to facilitate the preoperative individualized prediction of LN metastasis. Our nomogram performed well in both the training (AUC, 0.75; 95% CI, 0.68–0.82) and validation cohorts (AUC, 0.81; 95% CI, 0.69–0.94). Our nomogram also showed good calibration in both the training and validation cohorts.

To go beyond the purely mathematical measures of performance, such as the AUC, DCA was used to estimate the predicted net benefit of the model across all possible risk thresholds, thus making it easier to evaluate the effects of various risk thresholds [41, 42]. DCA showed that if the threshold probability is between 0.25 and 0.75, the current radiomics nomogram to predict LN metastases added more benefit than either the treat-all or treat-none scheme.

The current study has some limitations. The ROC value in the training cohort was lower than in the validation cohort. The study was lack of external validation of the model. Multicenter validation with a larger sample size is needed to acquire high-level evidence for clinical application. In addition, genetic markers have not yet been incorporated into our nomogram. Previous studies have showed that Smad4/DPDAC4 and MTA1 mRNA expression levels may be involved in the progression of PDAC, particularly in LN metastasis [43, 44]. A combination of gene marker panels and a radiomics signature may improve the ability to predict LN metastasis in patients with PDAC.

## Conclusion

Our radiomics nomogram, which is a noninvasive predictive tool that combines a radiomics signature with CT-reported LN status, shows favorable accuracy for preoperatively predicting LN metastasis in PDAC patients, especially in LN-positive patients.

## Supplementary Information

**Additional file 1: Supplementary 1.** Image preprocessing. **Supplementary 2.** Radiomics Features. **Supplementary 3.** the Least Absolute Shrinkage and Selection Operator (LASSO) Algorithm. **Supplementary 4.** Decision Curve Analysis (DCA). **Supplementary 5.** Radiomic Features Selected by LASSO Regularization.

## Data Availability

The datasets used and/or analyzed during the current study are available from the corresponding author on reasonable request.

## Abbreviations

LN: Lymph node
EUS-FNA: Endoscopic ultrasonography-guided fine-needle aspiration
MRI: Magnetic resonance imaging
MSCT: Multislice computed tomography
PDAC: Pancreatic ductal adenocarcinoma
HE: Hematoxylin and eosin
CA 19–9: Carbohydrate antigen 19–9
GLCM: Gray-level cooccurrence matrix
GLSZM: Gray-level size zone matrix
GLRLM: Gray-level run-length matrix
LASSO: Least absolute shrinkage and selection operator
ICC: Interobserver correlation coefficient
ROIs: Regions of interest
ROC: Receiver operating characteristic
AUC: Area under the curve
DCA: Decision curve analysis
TNM: Tumor–node–metastasis
CI: Confidence interval
CEA: Carcinoembryonic antigen
