# Supplementary material for: Radiomics nomogram for the preoperative prediction of lymph node metastasis in pancreatic ductal adenocarcinoma
Source: Cancer Imaging. 2022 Jan 6;22:4. doi: 10.1186/s40644-021-00443-1 (PMC8734356; doi:10.1186/s40644-021-00443-1)
Supplement: Supplementary file 1 — Additional file 1: Supplementary 1. Image preprocessing. Supplementary 2. Radiomics Features. Supplementary 3. the Least Absolute Shrinkage and Selection Operator (LASSO) Algorithm. Supplementary 4. Decision Curve Analysis (DCA). Supplementary 5. Radiomic Features Selected by LASSO Regularization. [file 40644_2021_443_MOESM1_ESM.docx]

**Radiomics Nomogram for the Preoperative Prediction of Lymph Node Metastasis in Pancreatic Ductal Adenocarcinoma**

**Authors**

Yun Bian, MD, PhD ^*^; Shiwei Guo, MD, PhD ^†^; Hui Jiang, MD, PhD ^‡^; Suizhi Gao ^†^; Chengwei Shao ^*^; Kai Cao, MD, PhD ^*^; Xu Fang ^*^; Jing Li, MD, PhD ^*^; Li Wang, MD, PhD ^*^; Chao Ma *; Jianming Zheng, MD, PhD ^‡^; Gang Jin, MD, PhD ^†^; Jianping Lu, MD, PhD ^*^

^*^Department of Radiology, Changhai Hospital, The Navy Military Medical University, Shanghai, China

^†^Department of Pancreatic Surgery, Changhai Hospital, The Navy Military Medical University, Shanghai, China

^‡^Department of Pathology, Changhai Hospital, The Navy Military Medical University, Shanghai, China

**Contents**

[Supplementary 1 Image preprocessing 3](#_Toc88861559)

[Supplementary 2 Radiomics Features 4](#_Toc88861560)

[Supplementary 3. the Least Absolute Shrinkage and Selection Operator (LASSO) Algorithm 17](#_Toc88861561)

[Supplementary 4. Decision Curve Analysis (DCA) 18](#_Toc88861562)

[Supplementary 5. Radiomic Features Selected by LASSO Regularization. 19](#_Toc88861563)

## Supplementary 1 Image preprocessing

Step 1- bias field correction: Differences in medical imaging factors can cause inconsistencies in the image intensity information from tissues of the same nature. We used the following formula for intensity normalization (where x represents the original intensity; f (x) indicates the normalized intensity; $\mu$ indicates the average value; $\delta$refers to variance; and s is an optional scaling ratio, which has been set to 1 by default). While retaining the intensity difference of the diagnostic value, the image intensity inconsistency caused by the difference in imaging parameters is reduced or even eliminated for subsequent imaging radiomics analysis.

Equation 1 (<https://pyradiomics.readthedocs.io/en/latest/radiomics.html#radiomics.imageoperations.normalizeImage>)

$$f\left( x \right)=\frac{s (x-u_{x})}{\delta_{x}}$$

Step 2- Resampling: All sets were isotropically resampled in all 3 dimensions via linear interpolation to ensure consistent voxel sizes and resolutions across all the machines and patients. As a result, the voxel dimensions of each case were 0.66 × 0.66 × 0.66 mm^3^. This step of processing was performed on all annotation masks as well to ensure that the masks remained in correspondence with the volumes.

## Supplementary 2 Radiomics Features

In this study we explored a feature-based approach to extract and quantify meaningful and reliable information from images. A total of 1029 quantitative imaging features were extracted. The methods of feature extraction used in this study included two categories: original feature classes and filter classes. Filter Classes included five categories: Wavelet, Square, Square Root, Logarithm, and Exponential. A total of 1029 2D and 3D features from primary tumors in each scan phase. The imaging traits were described in detail below.

**1.1 Firstorder**

Firstorder included 19 features.

| 1 | Energy | $energy=\sum_{i=1}^{N} ({X\left( i \right)+c)}^{2}$ |
| --- | --- | --- |
|  |  | Here, *c* is optional value, defined by “voxelArrayShift”, which shifts the intensities to prevent negative values in **X**. This ensures that voxels with the lowest gray values contribute the least to Energy, instead of voxels with gray level intensity closest to 0. |
| 2 | TotalEnergy | $\mathrm{TotalEnergy}=V_{voxel}\sum_{i=1}^{N} ({X\left( i \right)+c)}^{2}$ |
|  |  | Here, *c* is optional value, defined by “voxelArrayShift”, which shifts the intensities to prevent negative values in **X**. This ensures that voxels with the lowest gray values contribute the least to Energy, instead of voxels with gray level intensity closest to 0. |
| 3 | Entropy | $\mathrm{Entropy}=\sum_{i=1}^{N_{i}} p\left( i \right){log}_{2}(p\left( i \right)+\in)$ |
|  |  | Here, $\in$ is an arbitrarily small positive number (*≈* 2*:*2 *×* 10*^-^*^16^) |
| 4 | Minimum | *minimum* = min(**X**) |
| 5 | 10Percentile | $energy=\sum_{i=1}^{N} ({X\left( i \right)+c)}^{2}$ |
| 6 | 90Percentile | $energy=\sum_{i=1}^{N} ({X\left( i \right)+c)}^{2}$ |
| 7 | Maximum | *maximum* = max(**X**) |
| 8 | Mean | $\mathrm{mean}=\frac{1}{N}\sum_{i=1}^{N} X\left( i \right)$ |
| 9 | Median | The median gray level intensity within the ROI. |
| 10 | InterquartileRange | $\mathrm{InterquartileRange}=\boldsymbol{P}_{75}-\boldsymbol{P}_{25}$ |
|  |  | Here **P**25 and **P**75 are the 25*th* and 75*th* percentile of the image array, respectively. |
| 11 | Range | *range* = max(**X**) *-* min(**X**) |
| 12 | MeanAbsoluteDeviation (MAD) | $\mathrm{MAD}=\frac{1}{N}\sum_{i=1}^{N} \left\vert X\left( i \right)-\bar{X} \right\vert$ |
|  |  | Mean Absolute Deviation is the mean distance of all intensity values from the Mean Value of the image array. |
| 13 | RobustMeanAbsoluteDeviation (rMAD) | $\mathrm{rMAD}=\sum_{i=1}^{N} ({X\left( i \right)+c)}^{2}$ |
| 14 | RootMeanSquared (RMS) | $\mathrm{RMS}=\sqrt{\frac{1}{N}\sum_{i=1}^{N} ({X\left( i \right)+c)}^{2}}$ |
|  |  | Here, *c* is optional value, defined by “voxelArrayShift”, which shifts the intensities to prevent negative values in **X**. This ensures that voxels with the lowest gray values contribute the least to RMS, instead of voxels with gray level intensity closest to 0. |
| 15 | StandardDeviation | $\mathrm{StandardDeviation}=\sqrt{\frac{1}{N}\sum_{i=1}^{N} ({X\left( i \right)+\bar{X})}^{2}}$ |
| 16 | Skewness | $Skewness=\frac{\mu_{3}}{\sigma^{3}}=\frac{\frac{1}{N}\sum_{I=1}^{N} {(X\left( i \right)-\bar{X})}^{3}}{{(\sqrt{\frac{1}{N}\sum_{I=1}^{N} {(X\left( i \right)-\bar{X})}^{2}})}^{3}}$ |
|  |  | Where *µ*3 is the 3*rd* central moment. |
| 17 | Kurtosis | $Kurtosis=\frac{\mu_{4}}{\sigma^{4}}=\frac{\frac{1}{N}\sum_{I=1}^{N} {(X\left( i \right)-\bar{X})}^{4}}{{(\frac{1}{N}\sum_{I=1}^{N} {(X\left( i \right)-\bar{X})}^{2})}^{2}}$ |
|  |  | Where *µ*_4_ is the 4*^th^* central moment. |
| 18 | Variance | $\mathrm{Variance}=\frac{1}{N}\sum_{i=1}^{N} ({X\left( i \right)-\bar{X})}^{2}$ |
| 19 | Uniformity | $\mathrm{Uniformity}=\sum_{i=1}^{N_{I}} p({i)}^{2}$ |
| Notations:  **X** is an image of *N* voxels included in the ROI.  *Pi* is the first order histogram with *Nl* discrete intensity levels, in which *Nl* is the number of non-zero bins.  *pi* is the normalized first order histogram and equal to $\frac{P_{i}}{\sum P_{i}}$(This definition is the same for the following sections).  **10Percentile**  The 10*^th^* percentile of **X**.  **90Percentile**  The 90*^th^* percentile of **X** | | |

**1.2 Shape features**

Shape features describe the morphological property of the tumor region and were features rated from only the image without filtration. They included 13features.

| 1 | Volume | The volume of the ROI is approximated by multiplying the number of voxels in the ROI by the volume of a single voxel |
| --- | --- | --- |
| 2 | SurfaceArea | $\mathrm{SurfaceArea}=\sum_{i=1}^{N} \frac{1}{2}\left\vert a_{i}b_{i}{\times a}_{i}c_{i} \right\vert$ |
|  |  | N is the number of triangles forming the surface mesh of the volume (ROI) $a_{i}b_{i}$and$a_{i}c_{i}$ are the edges of the ith triangle formed by points $a_{i}$, $b_{i}$ and $c_{i}$  Surface Area is an approximation of the surface of the ROI in mm2, calculated using a marching cubes algorithm. |
| 3 | SurfaceVolumeRatio | $\mathrm{SurfaceVolumeRatio}=\frac{A}{V}$ |
|  |  | Here, a lower value indicates a more compact (sphere-like) shape. This feature is not dimension less, and is therefore (partly) dependent on the volume of the ROI. |
| 4 | Sphericity | $\mathrm{Sphericity}=\frac{\sqrt[3]{36\pi V^{2}}}{A}$ |
|  |  | Sphericity is a measure of the roundness of the shape of the tumor region relative to a sphere. It is a dimensionless measure, independent of scale and orientation.  The value range is 0 *< sphericity ≤* 1, where a value of 1 indicates a perfect sphere (a sphere has the smallest possible surface area for a given volume, compared to other solids). |
| 5 | Compactness1 | $Compactness1=\frac{v}{\sqrt{\pi A^{3}}}$ |
| 6 | Compactness2 | $Compactness2=36\pi\frac{v^{2}}{A^{3}}$ |
| 7 | SphericalDisproportion | $\mathrm{SphericalDisproportion}=\frac{A}{{4\pi R}^{2}}=\frac{v}{\sqrt[3]{36\pi{AV}^{2}}}$ |
| 8 | Maximum3DDiameter | Maximum 3D diameter is defined as the largest pairwise Euclidean distance between surface voxels in the ROI.  Also known as Feret Diameter. |
| 9 | Maximum2DDiameterColumn | Maximum 2D diameter (Column) is defined as the largest pairwise Euclidean distance between tumor surface voxels in the row-slice (usually the coronal) plane. |
| 10 | Maximum2DDiameterRow | Maximum 2D diameter (Row) is defined as the largest pairwise Euclidean distance between tumor surface voxels in the column-slice (usually the sagittal) plane |
| 11 | MajorAxis | The line through the foci is called the major axis, and the line perpendicular to it through the center is called the minor axis. Least axis is seen as the sum of the axis |
| 12 | MinorAxis |  |
| 13 | LeastAxis |  |
| 14 | Elongation | $\mathrm{Elongation}=\sqrt{\frac{\lambda_{minor}}{\lambda_{major}}}$ |
|  |  | Here, *λ*major and *λ*minor are the lengths of the largest and second largest principal component axes. The values range between 1 (where the cross section through the first and second largest principal moments is circle-like (non-elongated)) and 0 (where the object is a single point or 1 dimensional line). |
| 15 | Flatness | $\mathrm{Flatness}=\frac{\lambda_{least}}{\lambda_{major}}$ |
|  |  | Here, *λ*major and *λ*least are the lengths of the largest and smallest principal component axes. The values range between 1 (non-flat, sphere-like) and 0 (a flat object). |

**1.3 GLCM features**

GLCM features included 28 features

| 1 | Autocorrelation | $Autocorrelation=\sum_{i=1}^{N_{g}} \sum_{j=1}^{N_{g}} ijp(i,j)$ |
| --- | --- | --- |
| 2 | AverageIntensity | $\mu_{x}=\sum_{i=1}^{N_{g}} \sum_{j=1}^{N_{g}} p(i,j)i$ |
| 3 | ClusterProminence (CP) | $CP=\sum_{i=1}^{N_{g}} \sum_{j=1}^{N_{g}} {(i+j-u_{x}\left( i \right)-u_{y}\left( j \right))}^{4}p(i,j)$ |
| 4 | ClusterShade | $ClusterShade=\sum_{i=1}^{N_{g}} \sum_{j=1}^{N_{g}} {(i+j-u_{x}\left( i \right)-u_{y}\left( j \right))}^{3}p(i,j)$ |
| 5 | ClusterTendency (CT) | $CT=\sum_{i=1}^{N_{g}} \sum_{j=1}^{N_{g}} {(i+j-u_{x}\left( i \right)-u_{y}\left( j \right))}^{2}p(i,j)$ |
| 6 | Contrast | $Contrast=\sum_{i=1}^{N_{g}} \sum_{j=1}^{N_{g}} \left\vert i-j \right\vert^{2}p(i,j)$ |
| 7 | Correlation | $Correlation=\frac{\sum_{i=1}^{N_{g}} \sum_{j=1}^{N_{g}} p\left( i,j \right)ij-\mu_{x}(i)u_{y}\left( j \right)}{\sigma_{x}{\left( i \right)\sigma}_{y}\left( j \right)}$ |
| 8 | DifferenceAverage | $DifferenceAverage=\sum_{k=1}^{N_{g}-1} kp_{x-y}(k)$ |
| 9 | DifferenceEntropy | $DifferenceEntropy=\sum_{k=1}^{N_{g}-1} p_{x-y}(k){log}_{2}(p_{x-y}\left( k \right)+\epsilon)$ |
| 10 | DifferenceVariance | $DifferenceVariance=\sum_{k=1}^{N_{g}-1} kp_{x-y}(k)$ |
| 11 | Dissimilarity | $Dissimilarity=\sum_{i=1}^{N_{g}} \sum_{j=1}^{N_{g}} \left\vert i-j \right\vert p(i,j)$ |
| 12 | Energy | $Energy=\sum_{i=1}^{N_{g}} \sum_{j=1}^{N_{g}} \left[ p(i,j) \right]^{2}$ |
| 13 | Entropy | $Entropy=-\sum_{i=1}^{N_{g}} \sum_{j=1}^{N_{g}} p\left( i,j \right){log}_{2} \left[ p\left( i,j \right) \right]$ |
| 14 | Homofeaturesity1 | $Homogeneity=\sum_{i=1}^{N_{g}} \sum_{j=1}^{N_{g}} \frac{p(i,j)}{1+\left\vert i-j \right\vert}$ |
| 15 | Homofeaturesity2 | $Homogeneity=\sum_{i=1}^{N_{g}} \sum_{j=1}^{N_{g}} \frac{p(i,j)}{{1+\left\vert i-j \right\vert}^{2}}$ |
| 16 | Imc1 | $Imc1=\frac{HXY-HXY1}{max\{HX-HX\}}$ |
| 17 | Imc2 | $Imc2=\sqrt{1-e^{-2(HXY2-HXY)}}$ |
| 18 | Idm | $Idm=\sum_{i=1}^{N_{g}} \sum_{j=1}^{N_{g}} \frac{p(i,j)}{{1+\left\vert i-j \right\vert}^{2}},(i\neq j)$ |
| 19 | Idmn | $Idmn=\sum_{i=1}^{N_{g}} \sum_{j=1}^{N_{g}} \frac{p(i,j)}{1+(\frac{\left\vert i-j \right\vert^{2}}{N_{g}})}$ |
| 20 | Id | $Id=\sum_{i=1}^{N_{g}} \sum_{j=1}^{N_{g}} \frac{p(i,j)}{1+\left\vert i-j \right\vert}$ |
| 21 | Idn | $Idn=\sum_{i=1}^{N_{g}} \sum_{j=1}^{N_{g}} \frac{p(i,j)}{1+(\frac{\left\vert i-j \right\vert}{N_{g}})}$ |
| 22 | InverseVariance (IV) | $IV=\sum_{i=1}^{N_{g}} \sum_{j=1}^{N_{g}} \frac{p(i,j)}{\left\vert i-j \right\vert^{2}},(i\neq j)$ |
| 23 | MaximumProbability | $MaximumProbability=max(p\left( i,j \right))$ |
| 24 | SumAverage | $Sum average=\sum_{i=2}^{2N_{g}} \left[ ip_{x+y}(i) \right]$ |
| 25 | SumEntropy | $Sum entropy=-\sum_{i=2}^{2N_{g}} {Pp}_{x+y}(i){log}_{2} \left[ p_{x+y}(i) \right]$ |
| 26 | SumVariance | $Sum variance=\sum_{i=2}^{2N_{g}} {(i-SE)}^{2}p_{x+y}(i)$ |
| 27 | SumSquares | $Sum Squares=\sum_{i=1}^{N_{g}} \sum_{j=1}^{N_{g}} \left( i-\mu_{x} \right)^{2}p(i,j)$ |
| Notations:  **P**(*i; j*) is the co-occurence matrix for *δ* (distance) and *α* (angle)  *p*(*i; j*) is the normalized co-occurence matrix  *Ng* is the number of discrete intensity levels in the image  $p_{x}\left( i \right)=\sum_{j=1}^{N_{g}} P(i,j)$is the marginal row probabilities  $p_{xy}\left( j \right)=\sum_{j=1}^{N_{g}} P(i,j)$ is the marginal column probabilities  $u_{x}=\sum_{i=1}^{N_{g}} \sum_{j=1}^{N_{g}} P(i,j)i$ is the mean gray level intensity of *p_x_*  $u_{y}=\sum_{i=1}^{N_{g}} \sum_{j=1}^{N_{g}} P(i,j)j$ is the mean gray level intensity of *p_y_*  *σx* is the standard deviation of *px*  *σy* is the standard deviation of *py*  $p_{x+y}(k)=\sum_{i=1}^{N_{g}} \sum_{j=1}^{N_{g}} P(i,j)$, where *i* + *j* = *k*  $p_{x-y}(k)=\sum_{i=1}^{N_{g}} \sum_{j=1}^{N_{g}} P(i,j)$, where $\left\vert i-j \right\vert=k$  $HX=\sum_{i=1}^{N_{j}} p_{x}(i){log}_{2}(p_{x}\left( i \right)+\in)$ is the entropy of *p_x_*  $HY=\sum_{i=1}^{N_{j}} p_{y}(i){log}_{2}(p_{y}\left( i \right)+\in)$is the entropy of *p_y_*  $HXY=-\sum_{i=1}^{N_{g}} \sum_{j=1}^{N_{g}} p\left( i,j \right){log}_{2}\left( p\left( i,j \right)+\in\right)$is the entropy of *p*(*i; j*)  $HXY1=-\sum_{i=1}^{N_{g}} \sum_{j=1}^{N_{g}} p\left( i,j \right){log}_{2}\left( p\left( i,j \right)+\in\right)$  $\mathrm{HXY}2=-\sum_{i=1}^{N_{g}} \sum_{j=1}^{N_{g}} p_{x}(i)p_{y}(j){log}_{2}\left( p_{x}(i)p_{y}(j)+\in\right)$ | | |

**1.4 GLSZM features**

GLSZM features included 16 features

| 1 | SmallAreaEmphasis (SAE) | $SAE=\frac{\sum_{i=1}^{N_{g}} \sum_{j=1}^{N_{g}} \frac{\boldsymbol{P}(i,j)}{j^{2}}}{\sum_{i=1}^{N_{g}} \sum_{j=1}^{N_{g}} \boldsymbol{P}(i,j)）}$ |
| --- | --- | --- |
| 2 | LargeAreaEmphasis (LAE) | $LAE=\frac{\sum_{i=1}^{N_{g}} \sum_{j=1}^{N_{g}} \boldsymbol{P}(i,j)j^{2}}{\sum_{i=1}^{N_{g}} \sum_{j=1}^{N_{g}} \boldsymbol{P}(i,j)）}$ |
| 3 | GrayLevelNonUniformity (GLN) | $GLN=\frac{\sum_{i=1}^{N_{g}} {(\sum_{j=1}^{N_{g}} \boldsymbol{P}\left( i,j \right))}^{2}}{\sum_{i=1}^{N_{g}} \sum_{j=1}^{N_{g}} \boldsymbol{P}\left( i,j \right)}$ |
| 4 | GrayLevelNonUniformityNormalized (GLNN) | $GLNN=\frac{\sum_{i=1}^{N_{g}} {(\sum_{j=1}^{N_{g}} \boldsymbol{P}\left( i,j \right))}^{2}}{\sum_{i=1}^{N_{g}} \sum_{j=1}^{N_{g}} \boldsymbol{P}\left( i,j \right)^{2}}$ |
| 5 | SizeZoneNonUniformity (SZN) | $SZN=\frac{\sum_{i=1}^{N_{g}} {(\sum_{j=1}^{N_{g}} \boldsymbol{P}\left( i,j \right))}^{2}}{\sum_{i=1}^{N_{g}} \sum_{j=1}^{N_{g}} \boldsymbol{P}(i,j)}$ |
| 6 | SizeZoneNonUniformityNormalized (SZNN) | $SZNN=\frac{\sum_{i=1}^{N_{g}} {(\sum_{j=1}^{N_{g}} \boldsymbol{P}\left( i,j \right))}^{2}}{\sum_{i=1}^{N_{g}} \sum_{j=1}^{N_{g}} {\boldsymbol{P}(i,j)}^{2}}$ |
| 7 | ZonePercentage (ZP) | $ZP=\sum_{i=1}^{N_{g}} \sum_{j=1}^{N_{g}} \frac{\boldsymbol{P}(i,j)}{N_{p}}$ |
| 8 | GrayLevelVariance (GLV) | $GLV=\sum_{i=1}^{N_{g}} \sum_{j=1}^{N_{g}} \left( i-\mu\right)^{2}p(i,j)$ |
|  |  | $\mu=\sum_{i=1}^{N_{g}} \sum_{j=1}^{N_{g}} p(i,j)i$ |
| 9 | ZoneVariance (ZV) | $ZV=\sum_{i=1}^{N_{g}} \sum_{j=1}^{N_{g}} p(i,j)\left( j-\mu\right)^{2}$ |
| 10 | ZoneEntropy (ZE) | $ZE=\sum_{i=1}^{N_{g}} \sum_{j=1}^{N_{g}} p(i,j){log}_{2}(p\left( i,j \right)+\in)$ |
| 11 | LowGrayLevelZoneEmphasis (LGLZE) | $LGLZE=\frac{\sum_{i=1}^{N_{g}} \sum_{j=1}^{N_{s}} \frac{\boldsymbol{P}(i,j)}{i^{2}}}{\sum_{i=1}^{N_{g}} \sum_{j=1}^{N_{s}} P(i,j)}$ |
| 12 | HighGrayLevelZoneEmphasis (HGLZE) | $HGLZE=\frac{\sum_{i=1}^{N_{g}} {\sum_{j=1}^{N_{s}} \boldsymbol{P}\left( i,j \right)i}^{2}}{\sum_{i=1}^{N_{g}} \sum_{j=1}^{N_{s}} P(i,j)}$ |
| 13 | SmallAreaLowGrayLevelEmphasis (SALGLE) | $SALGLE=\frac{\sum_{i=1}^{N_{g}} \sum_{j=1}^{N_{s}} \frac{\boldsymbol{P}(i,j)}{i^{2}j^{2}}}{\sum_{i=1}^{N_{g}} \sum_{j=1}^{N_{s}} \boldsymbol{P}(i,j)}$ |
| 14 | SmallAreaHighGrayLevelEmphasis (SAHGLE) | $SAHGLE=\frac{\sum_{i=1}^{N_{g}} \sum_{j=1}^{N_{S}} \frac{\boldsymbol{P}(i,j)i^{2}}{j^{2}}}{\sum_{i=1}^{N_{g}} \sum_{j=1}^{N_{s}} \boldsymbol{P}(i,j)}$ |
| 15 | LargeAreaLowGrayLevelEmphasis (LALGLE) | $LALGLE=\frac{\sum_{i=1}^{N_{g}} \sum_{j=1}^{N_{s}} \frac{\boldsymbol{P}(i,j)j^{2}}{i^{2}}}{\sum_{i=1}^{N_{g}} \sum_{j=1}^{N_{s}} \boldsymbol{P}(i,j)）}$ |
| 16 | LargeAreaHighGrayLevelEmphasis (LAHGLE) | $LAHGLE==\frac{\sum_{i=1}^{N_{g}} {\sum_{j=1}^{N_{S}} \boldsymbol{P}\left( i,j \right)i}^{2}j^{2}}{\sum_{i=1}^{N_{g}} \sum_{j=1}^{N_{s}} \boldsymbol{P}(i,j)}$ |
| **Note:**  P (*i; j*) is the size zone matrix  *p* (*i; j*) is the normalized size zone matrix  *Ng* is the number of discrete intensity values in the image  *Ns* is the number of discrete zone sizes in the image  *Np* is the number of voxels in the image | | |

**2.5 GLRLM features**

GLRLM features included 16 features

| 1 | ShortRunEmphasis (SRE) | $SRE=\frac{\sum_{i=1}^{N_{g}} \sum_{j=1}^{N_{r}} \left[ \frac{\boldsymbol{P}(i,j\vert\theta)}{j^{2}} \right]}{\sum_{i=1}^{N_{g}} \sum_{j=1}^{N_{r}} \boldsymbol{P}(i,j\vert\theta)}$ |
| --- | --- | --- |
| 2 | LongRunEmphasis (LRE) | $LRE=\frac{\sum_{i=1}^{N_{g}} \sum_{j=1}^{N_{r}} j^{2}\boldsymbol{P}(i,j\vert\theta)}{\sum_{i=1}^{N_{g}} \sum_{j=1}^{N_{r}} \boldsymbol{P}(i,j\vert\theta)}$ |
| 3 | GrayLevelNonUniformity (GLNNN) | $GLNN=\frac{\sum_{i=1}^{N_{g}} \left[ \sum_{j=1}^{N_{r}} \boldsymbol{P}(i,j\vert\theta) \right]^{2}}{\sum_{i=1}^{N_{g}} \sum_{j=1}^{N_{r}} \boldsymbol{P}(i,j\vert\theta)}$ |
| 4 | GrayLevelNonUniformityNormalized (RLNN) | $RLNN=\frac{\sum_{i=1}^{N_{r}} {(\sum_{j=1}^{N_{g}} \boldsymbol{P}\left( i,j \vert\theta\right)}^{2}}{\sum_{i=1}^{N_{g}} \sum_{j=1}^{N_{r}} \boldsymbol{P}(i,j\vert\theta)}$ |
| 5 | RunLengthNonUniformity (RLN) | $RLN=\frac{\sum_{j=1}^{N_{r}} {\sum_{i=1}^{N_{g}} \boldsymbol{(P}(i,j\vert\theta))}^{2}}{\sum_{i=1}^{N_{g}} \sum_{j=1}^{N_{r}} \boldsymbol{P}(i,j\vert\theta)}$ |
| 6 | RunLengthNonUniformityNormalized (RLNN) | $RLNN=\frac{\sum_{j=1}^{N_{r}} {(\sum_{i=1}^{N_{g}} \boldsymbol{P}(i,j\vert\theta))}^{2}}{\sum_{i=1}^{N_{g}} \sum_{j=1}^{N_{r}} \boldsymbol{P}(i,j\vert\theta)}$ |
|  |  | RLNN measures the similarity of run lengths throughout the image, with a lower value indicating more homofeaturesity among run lengths in the image. This is the normalized version of the RLN formula |
| 7 | RunPercentage (RP) | $RP=\sum_{i=1}^{N_{g}} \sum_{j=1}^{N_{r}} \frac{\boldsymbol{P}(i,j\vert\theta)}{N_{p}}$ |
| 8 | GrayLevelVariance (GLV) | $GLV=\sum_{i=1}^{N_{g}} \sum_{j=1}^{N_{g}} p\left( i,j \vert\theta\right))\left( i-\mu\right)^{2}$ |
|  |  | Here, $\mu=\sum_{i=1}^{N_{g}} \sum_{j=1}^{N_{r}} p\left( i,j \vert\theta\right))i$  GLV measures the variance in gray level intensity for the runs. |
| 9 | RunVariance (RV) | $RV=\sum_{i=1}^{N_{g}} \sum_{j=1}^{N_{r}} p\left( i,j \vert\theta\right))\left( j-\mu\right)^{2}$ |
|  |  | Here, $\mu=\sum_{i=1}^{N_{g}} \sum_{j=1}^{N_{r}} p\left( i,j \vert\theta\right))j$  RV is a measure of the variance in runs for the run lengths. |
| 10 | RunEntropy (RE) | $RE=\sum_{i=1}^{N_{g}} \sum_{j=1}^{N_{g}} p\left( i,j \vert\theta\right){log}_{2}(p\left( i,j \vert\theta\right)+\epsilon)$ |
|  |  | Here, $\epsilon$ is an arbitrarily small positive number (*≈* 2*:*2 *×* 10*^-^*^16^).  RE measures the uncertainty/randomness in the distribution of run lengths and gray levels. A higher value indicates more heterofeaturesity in the texture patterns |
| 11 | LowGrayLevelRunEmphasis (LGLRE) | $LGLRE=\frac{\sum_{i=1}^{N_{g}} \sum_{j=1}^{N_{r}} \frac{\boldsymbol{P}(i,j\vert\theta)}{i^{2}}}{\sum_{i=1}^{N_{g}} \sum_{j=1}^{N_{r}} \boldsymbol{P}(i,j\vert\theta)}$ |
| 12 | HighGrayLevelRunEmphasis (HGLRE) | $HGLRE=\frac{\sum_{i=1}^{N_{g}} \sum_{j=1}^{N_{r}} \boldsymbol{P}(i,j\vert\theta)i^{2}}{\sum_{i=1}^{N_{g}} \sum_{j=1}^{N_{r}} \boldsymbol{P}(i,j\vert\theta)}$ |
| 13 | ShortRunLowGrayLevelEmphasis (SRLGLE) | $\mathrm{SRLGLE}=\frac{\sum_{i=1}^{N_{g}} \sum_{j=1}^{N_{r}} \boldsymbol{P}\left( i,j \vert\theta\right)i^{2}j^{2}}{\sum_{i=1}^{N_{g}} \sum_{j=1}^{N_{r}} \boldsymbol{P}(i,j\vert\theta)}$ |
| 14 | ShortRunHighGrayLevelEmphasis (SRHGLE) | $SRHGLE=\frac{\sum_{i=1}^{N_{g}} \sum_{j=1}^{N_{r}} \frac{\boldsymbol{P}\left( i,j \vert\theta\right)e^{2}}{j^{2}}}{\sum_{i=1}^{N_{g}} \sum_{j=1}^{N_{r}} \boldsymbol{P}(i,j\vert\theta)}$ |
| 15 | LongRunLowGrayLevelEmphasis (LRLGLE) | $LRLGLE=\frac{\sum_{i=1}^{N_{g}} \sum_{j=1}^{N_{r}} \frac{\boldsymbol{P}\left( i,j \vert\theta\right)}{i^{2}j^{2}}}{\sum_{i=1}^{N_{g}} \sum_{j=1}^{N_{r}} \boldsymbol{P}(i,j\vert\theta)}$ |
| 16 | LongRunHighGrayLevelEmphasis (LRHGLE) | $LRHGLE=\frac{\sum_{i=1}^{N_{g}} \sum_{j=1}^{N_{r}} \boldsymbol{P}\left( i,j \vert\theta\right)i^{2}j^{2}}{\sum_{i=1}^{N_{g}} \sum_{j=1}^{N_{r}} \boldsymbol{P}(i,j\vert\theta)}$ |
| Notations:  $\boldsymbol{P}\left( i,j \vert\theta\right)$ is the run length matrix of direction *θ*  $p\left( i,j \vert\theta\right)$ is the normalized run length matrix  *Ng* is the number of discrete intensity values in the image  *Nr* is the number of discrete run lengths in the image  *Np* is the number of voxels in the image | | |

|  | Table 1 1029 Radiomics Feathers | | | | |
| --- | --- | --- | --- | --- | --- |
|  | **Firstorder** | **Shape** | **GLCM** | **GLSZM** | **GLRLM** |
| 1 | Energy | Volume | Autocorrelation | SmallAreaEmphasis | ShortRunEmphasis |
| 2 | TotalEnergy | SurfaceArea | AverageIntensity | LargeAreaEmphasis | LongRunEmphasis |
| 3 | Entropy | SurfaceVolumeRatio | ClusterProminence | GrayLevelNonUniformity | GrayLevelNonUniformity |
| 4 | Minimum | Sphericity | ClusterShade | GrayLevelNonUniformityNormalized | GrayLevelNonUniformityNormalized |
| 5 | 10Percentile | Compactness1 | ClusterTendency | SizeZoneNonUniformity | RunLengthNonUniformity |
| 6 | 90Percentile | Compactness2 | Contrast | SizeZoneNonUniformityNormalized | RunLengthNonUniformityNormalized |
| 7 | Maximum | SphericalDisproportion | Correlation | ZonePercentage | RunPercentage |
| 8 | Mean | Maximum3DDiameter | DifferenceAverage | GrayLevelVariance | GrayLevelVariance |
| 9 | Median | Maximum2DDiameterColumn | DifferenceEntropy | ZoneVariance | RunVariance |
| 10 | InterquartileRange | Maximum2DDiameterRow | DifferenceVariance | ZoneEntropy | RunEntropy |
| 11 | Range | MajorAxis | Dissimilarity | LowGrayLevelZoneEmphasis | LowGrayLevelRunEmphasis |
| 12 | MeanAbsoluteDeviation | MinorAxis | Energy | HighGrayLevelZoneEmphasis | HighGrayLevelRunEmphasis |
| 13 | RobustMeanAbsoluteDeviation | LeastAxis | Entropy | SmallAreaLowGrayLevelEmphasis | ShortRunLowGrayLevelEmphasis |
| 14 | RootMeanSquared | Elongation | Homofeaturesity1 | SmallAreaHighGrayLevelEmphasis | ShortRunHighGrayLevelEmphasis |
| 15 | StandardDeviation | Flatness | Homofeaturesity2 | LargeAreaLowGrayLevelEmphasis | LongRunLowGrayLevelEmphasis |
| 16 | Skewness |  | Imc1 | LargeAreaHighGrayLevelEmphasis | LongRunHighGrayLevelEmphasis |
| 17 | Kurtosis |  | Imc2 |  |  |
| 18 | Variance |  | Idm |  |  |
| 19 | Uniformity |  | Idmn |  |  |
| 20 |  |  | Id |  |  |
| 21 |  |  | Idn |  |  |
| 22 |  |  | InverseVariance |  |  |
| 23 |  |  | MaximumProbability |  |  |
| 24 |  |  | SumAverage |  |  |
| 25 |  |  | SumEntropy |  |  |
| 26 |  |  | SumVariance |  |  |
| 27 |  |  | SumSquares |  |  |

**Reference:** van Griethuysen JJ, Fedorov A, Parmar C, Hosny A, Aucoin N, Narayan V, Beets-Tan RG, Fillion-Robin J-C, Pieper S, Aerts HJJCr: Computational radiomics system to decode the radiographic phenotype. 2017;77:e104-e107

## Supplementary 3. the Least Absolute Shrinkage and Selection Operator (LASSO) Algorithm

LASSO is a powerful algorithm for regression analysis with high dimensional predictors. In our study, the LASSO algorithm was combined with the logistic regression model for model development. We used the LASSO logistic regression model to select the most important predictive features and construct a radiomics signature in the training set. This algorithm minimizes a log partial likelihood subject to the sum of the absolute values of the parameters bounded by a constant:

$\hat{\beta}=arg min\mathcal{l}\left( \beta\right)$, subject to $\sum\left| \beta_{j} \right|\leq t$

where $\hat{\beta}$ is the obtained parameters, $\mathcal{l}\left( \beta\right)$ is the log partial likelihood of the logistic regression model, and $t＞0$ is a constant.

The LASSO algorithm shrinks some coefficients and reduces others to exactly 0 via the absolute constraint. Thus, LASSO is an outstanding method for feature selection by retaining the good features of both subset selection and ridge regression.

**Reference:** Tibshirani R. Regression shrinkage and selection via the lasso. Journal of the Royal Statistical Society Series B (Methodological) 1996:267-88

## Supplementary 4. Decision Curve Analysis (DCA)

In our study, the DCA method was used to evaluate the clinical utility of the radiomics nomogram. The DCA algorithm assesses prediction models by calculating the range of threshold probabilities in which a prediction or prognostic model was clinically useful. DCA is a compositive method for evaluating and comparing different diagnostic and prognostic models. The theory of DCA can be illustrated by the equation below:

$$\frac{a-c}{d-b}=\frac{1-P_{t}}{P_{t}}$$

where *d* – *b* represents the influence of unnecessary treatment. If treatment is directed by a prediction model, *d* – *b* is the harm related to a false-positive result compared with a true-negative result. Inversely, *a* – *c* represents the consequence of rejecting beneficial treatment, in other words, the harm from a false-negative result compared with a true-positive result. *P_t_* represents where the expected benefit of treatment is equal to the expected benefit of refraining from treatment.

**Reference:** Vickers AJ, Elkin EB. Decision curve analysis: a novel method for evaluating prediction models. Med Decis Making. 2006 Nov-Dec;26(6):565-74.

## Supplementary 5. Radiomic Features Selected by LASSO Regularization.

A total of 1029 radiomics features from the arterial phase of CT were extracted and grouped on the basis of the LN metastasis. Consequently, the radiomics characteristics were reduced to 13 features. There was a significant difference in arterial rad-scores between the LN-positive and the LN-negative patients (p =0.002).


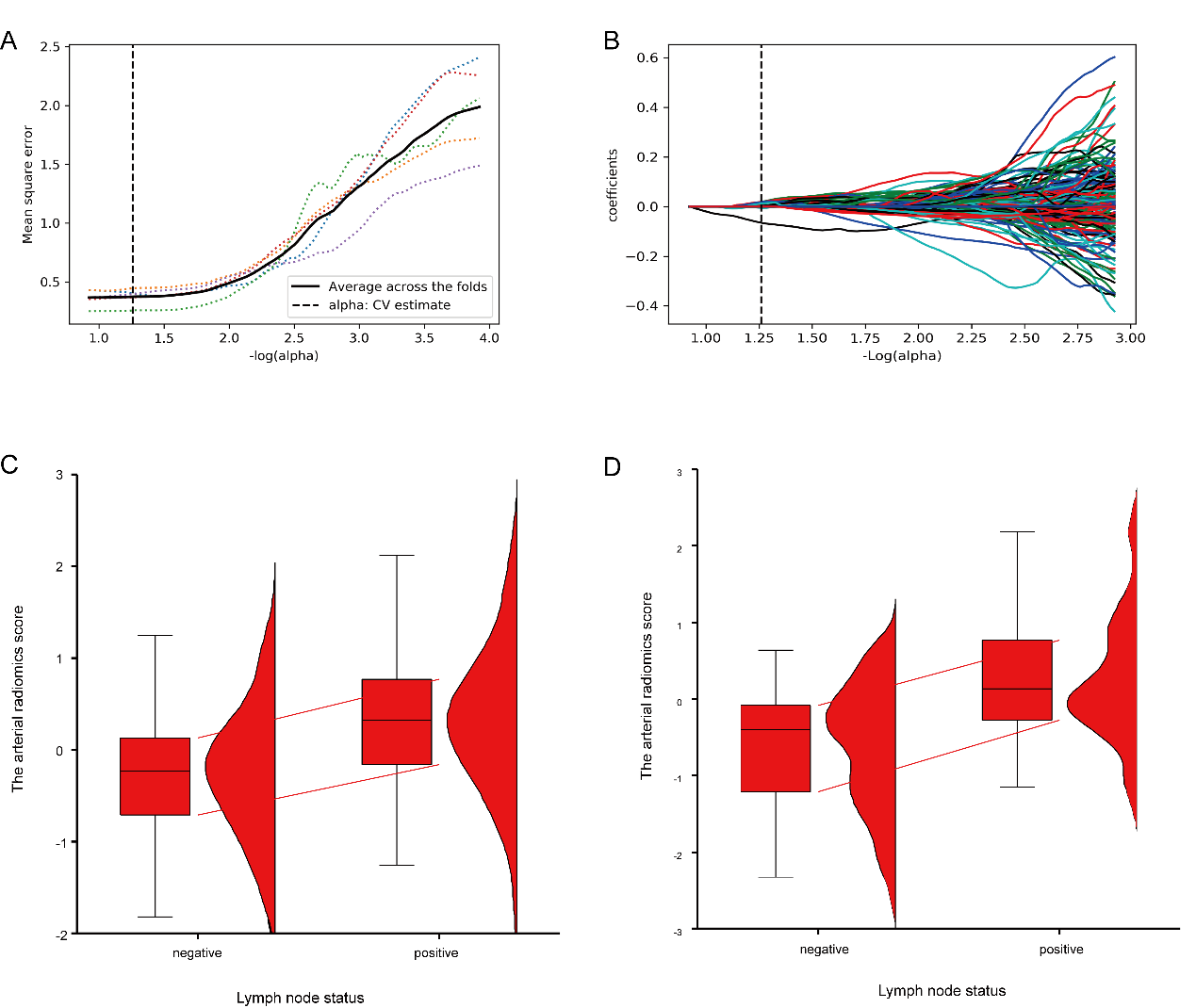


**Figure A, B** Radiomic feature selection by using a parametric method, the least absolute shrinkage and selection operator (LASSO). **(A)** Selection of the tuning parameter (λ) in the LASSO model via 5-fold cross validation based on minimum criteria. Mean square error from the LASSO regression cross-validation procedure were plotted as a function of -log(λ). The y-axis indicates mean square error. The lower x-axis indicates the -log(λ). Black line indicate average square error values for each model with a given λ, and colorful lines show mean square error of λ value. The vertical black lines define the optimal values of λ, where the model provides its best fit to the data. The optimal λ value of 0.055 with log(λ) = -1.26 was selected. **(B)** LASSO coefficient profiles of the 1024 texture features. The dotted vertical line was plotted at the value selected using 5-fold cross-validation in A. The thirteen resulting features with nonzero coefficients are indicated in the plot. **C, D** Combo chart of the arterial radiomics scores by the LASSO regression formula. Combo chart included box plot and density plot. 25^th^ and 75^th^ percentiles connecting lines between groups. (**C)** training cohort, (**D)** validation cohort.
